# Postoperative cystoid macular edema after pars plana vitrectomy for epiretinal membrane: incidence, anatomical and functional outcomes, and response to intravitreal dexamethasone implant

**DOI:** 10.1186/s40942-026-00888-1

**Published:** 2026-06-23

**Authors:** Leonardo Provetti Cunha, Manuela Ferreira Guimarães, Leandro Cabral Zacharias, Rony Carlos Preti, Luciana Virgínia Ferreira Costa-Cunha, Mário Luiz Ribeiro Monteiro

**Affiliations:** 1 Department of Ophthalmology, Federal University of Juiz de Fora, Juiz de Fora, Minas Gerais Brazil; 2 Hospital de Olhos Juiz de Fora, Av. Barão do Rio Branco, 4051, Bom Pastor, Juiz de Fora, Minas Gerais 36021-660 Brazil; 3 Department of Ophthalmology, University of São Paulo, São Paulo, Brazil; 4 Faculdade de Ciências Médicas e da Saúde de Juiz de Fora, Juiz de Fora, Minas Gerais Brazil

**Keywords:** Cystoid macular edema, Pars plana vitrectomy, Epiretinal membrane, Dexamethasone implant, Ozurdex, Optical coherence tomography, Central macular thickness

## Abstract

**Background:**

To determine the incidence of postoperative cystoid macular edema (CME) following pars plana vitrectomy (PPV) with epiretinal membrane (ERM) peeling, and to evaluate the anatomical and functional response to intravitreal dexamethasone implant (Ozurdex; AbbVie) in affected eyes.

**Methods:**

This retrospective, single-center cohort study included 92 consecutive eyes with idiopathic ERM that underwent PPV with ERM peeling by a single surgeon. Eyes were allocated to a control group (*n* = 84), comprising eyes without postoperative CME, or to a CME group (*n* = 8), comprising eyes that developed postoperative CME confirmed on swept-source optical coherence tomography (SS-OCT) and subsequently received intravitreal dexamethasone implant (DEX). Best-corrected visual acuity (BCVA, logMAR), central macular thickness (CMT, µm), and intraocular pressure (IOP, mmHg) were assessed preoperatively and postoperatively in both groups, and before and after DEX implantation in the CME group.

**Results:**

The incidence of postoperative CME requiring treatment was 8.7%. Most baseline characteristics were comparable between groups, although preoperative IOP was significantly lower in the CME group. CMT decreased significantly after surgery in both the control group (335.1 ± 37.3 to 289.5 ± 22.9 μm; *p* < 0.001) and the CME group (382.9 ± 74.6 to 310.9 ± 22.9 μm; *p* = 0.033); however, postoperative CMT remained significantly higher in the CME group than in controls (*p* = 0.034). DEX implantation produced an additional significant CMT reduction (379.9 ± 67.1 to 302.7 ± 35.3 μm; *p* = 0.003), reducing the between-group difference to a non-significant level (*p* = 0.332). BCVA improved significantly after surgery in the control group (0.32 ± 0.26 to 0.04 ± 0.10 logMAR; *p* < 0.001) but not in the CME group (*p* = 0.582). Following DEX implantation, BCVA improved significantly in the CME group (0.47 ± 0.35 to 0.22 ± 0.25 logMAR; *p* = 0.002), approaching values observed in the control group (*p* = 0.078). IOP remained stable in controls (*p* = 0.576). A transient but significant IOP increase was observed in the CME group after surgery (11.5 ± 2.7 to 15.3 ± 3.7 mmHg; *p* = 0.019), with no further change after DEX implantation (*p* = 0.747).

**Conclusions:**

Postoperative CME requiring treatment occurred in approximately 1 in 11 eyes following PPV with ERM peeling and was associated with significant anatomical and functional impairment. The present findings suggest that intravitreal dexamethasone implant was associated with improvement in selected cases of postoperative CME without additional impact on IOP; however, larger prospective studies are required to confirm these results.

## Background

Macular epiretinal membrane (ERM) is characterized by the proliferation of fibrocellular tissue on the inner retinal surface, generating tangential tractional forces that lead to macular distortion, contraction, and increased retinal thickness [1]. Pars plana vitrectomy (PPV) with membrane peeling has been widely established as an effective surgical approach for its management [2, 3]. Although visual acuity typically improves after surgery, a subset of patients continues to experience persistent visual disturbances, including blurred vision, scotomas, and metamorphopsia. Postoperative visual outcomes remain variable and are influenced by multiple factors, such as disease stage, baseline visual acuity, patient age, symptom duration, and the integrity of both inner and outer retinal layers [4–9].

Optical coherence tomography (OCT) has become the primary imaging modality for evaluating retinal structural changes in both preoperative and postoperative settings [5]. It plays a central role in assessing disease severity, estimating visual prognosis, and monitoring retinal recovery after surgery. Although increased macular thickness has been associated with greater disease severity and worse visual outcomes [10, 11], it does not fully account for the variability in functional recovery. Consequently, increasing attention has been directed toward additional OCT-derived biomarkers that may better reflect retinal integrity and functional prognosis — including disorganization of the retinal inner layers (DRIL), alterations of the outer retinal layers, dissociated optic nerve fiber layer (DONFL), and intraretinal microcystic changes [11–14]. Among these, cystoid macular edema (CME) occupies a distinct position: unlike the structural abnormalities that are largely permanent, CME is a potentially reversible condition and, therefore, a relevant therapeutic target.

In a recent study, we demonstrated that patients undergoing PPV for ERM removal, despite achieving significant visual improvement, exhibited reduced retinal sensitivity on microperimetry compared with healthy controls [15]. The presence of DRIL, outer retinal layer alterations, and postoperative microcystic macular edema were all associated with more limited visual recovery [16, 17]. These findings reinforced the clinical importance of identifying and managing modifiable postoperative complications — particularly CME, which has been reported in a variable proportion of eyes following ERM peeling and may contribute to delayed or incomplete functional recovery.

Intravitreal corticosteroids have been proposed as adjuvant treatment for CME after ERM peeling, given their anti-inflammatory and anti-permeability effects. However, current evidence remains inconsistent, with some studies demonstrating anatomical and functional benefits and others failing to show significant improvement [18–20]. These discrepancies likely reflect heterogeneity in study design, patient selection, baseline retinal damage, timing of intervention, and differences among corticosteroid formulations with respect to pharmacokinetics and duration of action [21, 22]. 

Among available options, the dexamethasone intravitreal implant (DEX; Ozurdex^®^, AbbVie, North Chicago, IL, USA) may offer particular advantages due to its sustained-release profile, which provides prolonged anti-inflammatory effects and stabilization of the blood–retinal barrier over several months [22]. Despite its theoretical appeal, evidence regarding DEX implant efficacy in this specific context remains limited and conflicting: some reports suggest anatomical benefit without consistent functional gains, while others demonstrate improvements in both outcomes, making it difficult to draw firm conclusions [17, 20, 21]. Prospective studies with standardized outcome measures are needed to better characterize the role of DEX implant in this setting.

The aims of this study were as follows: (1) to determine the incidence of postoperative cystoid macular edema following PPV with ERM peeling; and (2) to evaluate the anatomical and functional response to intravitreal dexamethasone implant in eyes that developed this complication.

## Methods

This retrospective, single-center cohort study was conducted at the Hospital de Olhos Juiz de Fora, Minas Gerais, Brazil. The study was approved by the Institutional Review Board of the Universidade Federal de Juiz de Fora (CEP/UFJF; approval number 3.919.662; CAAE: 12296919.0.3002.0065) and conducted in accordance with the tenets of the Declaration of Helsinki. Given its retrospective nature, informed consent was waived by the ethics committee. Eyes in the CME group received additional individual written informed consent prior to the off-label use of the intravitreal dexamethasone implant (Ozurdex; AbbVie), as its approved indications in Brazil (ANVISA) are limited to diabetic macular edema, retinal vein occlusion, and non-infectious posterior uveitis.

### Study population and eligibility

The study included consecutive eyes that underwent pars plana vitrectomy (PPV) combined with idiopathic epiretinal membrane (ERM) peeling between January 2020 and December 2024. The minimum follow-up period required for inclusion was 12 months. All procedures were performed by a single surgeon (LPC) at the Hospital de Olhos Juiz de Fora, ensuring procedural consistency across cases.

#### Inclusion criteria

eyes with idiopathic ERM confirmed on SS-OCT and clinical examination, undergoing PPV with ERM and ILM peeling, with a minimum postoperative follow-up of 12 months.

#### Exclusion criteria

secondary ERM (associated with prior retinal detachment surgery, retinal vein occlusion, diabetic retinopathy, uveitis, laser photocoagulation, or any prior intraocular surgery other than uncomplicated phacoemulsification); preoperative intraocular pressure (IOP) greater than 21 mmHg; use of any topical antiglaucoma medication; clinical or structural signs of glaucomatous optic neuropathy (abnormal optic nerve head morphology on fundoscopy or OCT retinal nerve fiber layer thinning consistent with glaucoma); diabetes mellitus; and preoperative intraretinal cystic or schisis-like changes on SS-OCT.

### Group allocation

Eyes were allocated to one of two groups based on their postoperative course. The control group comprised eyes that completed 12 months of follow-up without developing any OCT-confirmed intraretinal cystic changes. The CME group comprised eyes that developed postoperative CME persisting despite standardized topical treatment and subsequently underwent DEX implantation. The final analytic sample included 92 eyes: 84 in the control group and 8 in the CME group.

### Definition of postoperative CME and refractory CME

**Fig. 1:**
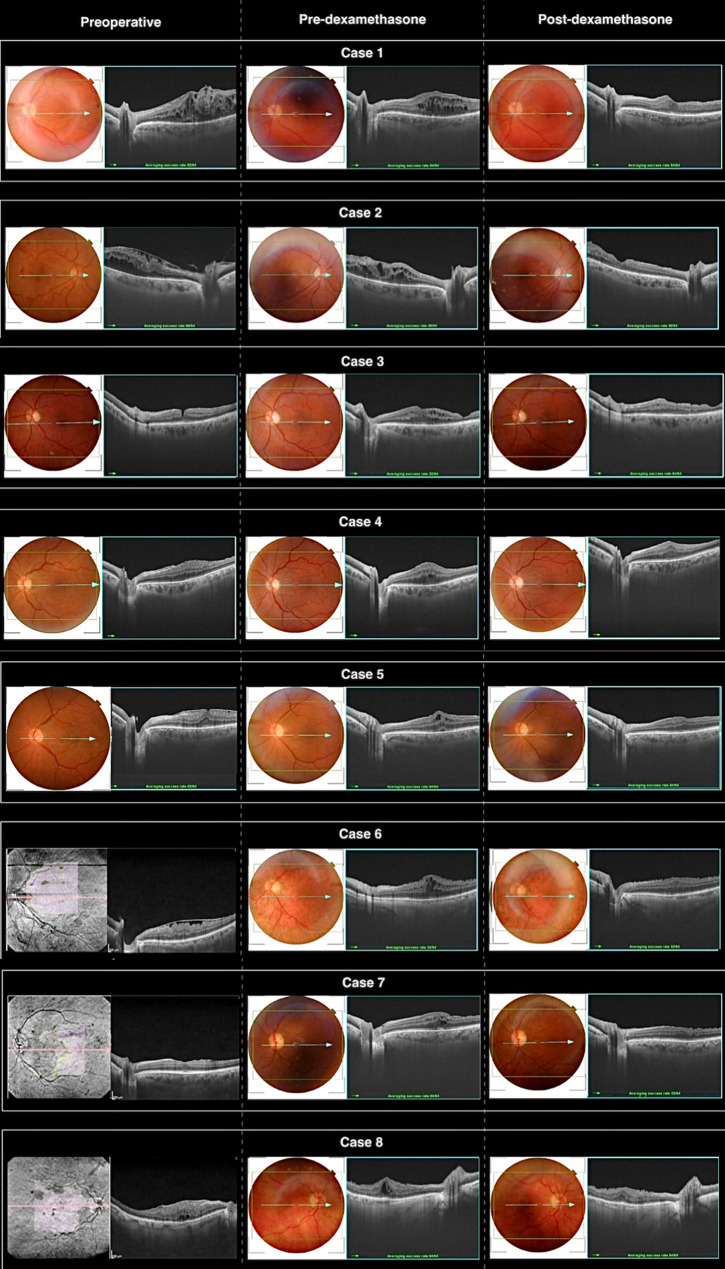
Representative optical coherence tomography images of eight eyes with cystoid macular edema after pars plana vitrectomy and epiretinal membrane peeling treated with intravitreal dexamethasone implant. Columns correspond to preoperative (Pre-operative), postoperative before dexamethasone implantation (pre-dexamethasone), and post-treatment (post-dexamethasone) time points. Images demonstrate postoperative CME development and subsequent anatomical response following dexamethasone therapy

Postoperative CME was defined as the de novo presence of intraretinal hyporeflective cystic spaces within the inner nuclear layer and/or the outer plexiform layer, associated or not with subretinal fluid, identified on SS-OCT at any point within 12 months following surgery (Fig. 1). Inclusion in the CME group additionally required evidence of increased macular thickness relative to the earliest postoperative examination, thereby confirming true structural worsening with the emergence of intraretinal cystic spaces compared with the post-peeling baseline. All eyes in the CME group had a central macular thickness greater than 300 μm at the time of CME diagnosis (Table 2). Eyes in the control group were required to show no macular thickening beyond the postoperative baseline at any follow-up visit. No eye included in the study presented with preoperative intraretinal cystic or schisis-like changes on SS-OCT. Degenerative microcystic changes without evidence of progressive retinal thickening relative to the earliest postoperative examination were not classified as postoperative CME and were not included in the CME group.

**Refractory CME** was defined as the persistence of intraretinal hyporeflective cystic spaces with a central macular thickness greater than 300 μm on SS-OCT following a minimum of 6 weeks of topical ketorolac 0.5% (one drop every 8 h) and prednisolone acetate 1% (one drop every 6 h). DEX implantation was performed only after SS-OCT confirmed persistent CME with no anatomical improvement despite this topical regimen. In the CME group, CME was diagnosed on SS-OCT between 4 and 8 weeks before DEX implantation in all eyes, with this interval corresponding to the duration of the mandatory topical treatment trial prior to the decision to escalate to intravitreal therapy.

### Surgical technique

All procedures were performed by a single surgeon (L.P.C.) at the Juiz de Fora Eye Hospital under local anesthesia with intravenous sedation and retrobulbar block. A 25-gauge three-port pars plana vitrectomy system with a 7,500 cuts-per-minute vitrectomy probe (Constellation Vision System; Alcon, Fort Worth, TX, USA) was used in all cases. Intraoperative triamcinolone acetonide (4 mg/0.1 mL) was administered in all eyes prior to membrane peeling to facilitate visualization of the posterior hyaloid and residual vitreous cortex. The epiretinal membrane and internal limiting membrane (ILM) were simultaneously stained using Membrane Blue Dual (D.O.R.C., Zuidland, the Netherlands), a combined solution of Brilliant Blue G and trypan blue. The ERM was grasped and peeled using Eckardt end-gripping forceps, followed by ILM peeling encompassing approximately two disc diameters centered on the fovea. ILM peeling was performed in all 92 eyes. In all eight CME group eyes, the posterior hyaloid was found to be spontaneously detached from the macula intraoperatively, precluding the need for surgical induction of posterior vitreous detachment (PVD). Air was used as the tamponade agent at the conclusion of all procedures. The uniform use of intraoperative triamcinolone across all eyes means that this variable cannot account for the differential occurrence of postoperative CME in the affected group.

### Follow-up protocol

Eyes in the CME group were followed according to the same predefined schedule as controls. Postoperative visits — including comprehensive ophthalmic examination and SS-OCT — were scheduled at postoperative day 1, week 1, and months 1, 3, 6, and 12. Unscheduled visits could occur earlier in cases of visual deterioration or symptom worsening. Following DEX implantation, CME group eyes continued follow-up according to the same schedule until completing at least 12 months of follow-up, or more frequently when clinically indicated.

### Variables and outcomes

The following variables were analyzed: age, sex, laterality, lens status, glaucoma status, systemic comorbidities (hypertension, renal disease), time interval between surgery and DEX implantation, intraocular pressure (IOP), best-corrected visual acuity (BCVA) converted to logMAR, and central macular thickness (CMT) in microns as measured by SS-OCT. Baseline comparisons between groups included age, sex, lens status, preoperative IOP, preoperative BCVA, and preoperative CMT. Longitudinal comparisons in the control group were performed between preoperative and 12-month postoperative values of BCVA, CMT, and IOP. In the CME group, comparisons were performed between values obtained at the time of CME diagnosis — immediately prior to DEX implantation — and at the final follow-up visit after treatment.

### Statistical analysis

Continuous variables are presented as mean ± standard deviation. Normality was assessed using the Shapiro-Wilk test. Given the small sample size of the CME group (*n* = 8), in which the Shapiro-Wilk test has limited power to detect departures from normality, both parametric and non-parametric tests were performed in parallel for all CME group analyses. Between-group comparisons of continuous variables were performed using Student’s t-test or Welch’s t-test (for unequal variances) or the Mann-Whitney U test, as appropriate. Within-group longitudinal comparisons were performed using the paired t-test or the Wilcoxon signed-rank test, as appropriate. Results of non-parametric analyses were consistent with parametric findings throughout. Effect sizes (Cohen’s d) are reported for within-group paired comparisons. Confidence intervals are provided where applicable. No multivariate analysis was performed due to the limited number of CME cases, which precluded meaningful multivariable modeling. Given the exploratory and descriptive nature of the study, no formal correction for multiple comparisons was applied; exact p-values are reported throughout, and the potential for inflated type I error is acknowledged as a limitation. A p-value < 0.05 was considered statistically significant. All analyses were performed using SPSS software (version 22; IBM Corp., Armonk, NY, USA).

## Results

### Baseline characteristics

A total of 92 consecutive eyes were included in the study, of which 84 (91.3%) were classified as controls and 8 (8.7%) as the CME group. All 92 eyes presented with idiopathic ERM; no secondary ERMs were included. No patient in either group had a diagnosis of glaucoma, diabetes mellitus, or was receiving topical antiglaucoma medication. Systemic hypertension and chronic renal disease were present in a minority of patients in both groups, with no significant between-group difference. The mean age of the overall cohort was 71.91 ± 8.35 years, with no statistically significant difference between the control group (71.80 ± 8.47 years) and the CME group (73.12 ± 7.22 years; *p* = 0.64) (Table 1). Regarding sex distribution, females accounted for 54.3% of the total sample, with a higher proportion in the control group (57.1%) compared to the CME group (25.0%), although this difference did not reach statistical significance (*p* = 0.09). In terms of lens status, the majority of eyes were pseudophakic or had undergone combined phacoemulsification and pars plana vitrectomy (Phaco + PPV), representing 40.2% and 53.3% of the total sample, respectively. A smaller proportion of eyes were phakic (6.5%). The distribution of lens status was comparable between groups (*p* = 0.58, Fisher’s exact test). Preoperative IOP was significantly lower in the CME group (11.50 ± 2.67 mmHg) compared to controls (14.06 ± 2.88 mmHg; *p* = 0.027, Welch’s t-test), a finding discussed further in the context of postoperative IOP responses.

**Table 1:** Baseline characteristics of all participants included in the study

Variable	Category	Controls	CME group	Total	*p*-value
Age (years)	mean ± SD	71.80 ± 8.47	73.12 ± 7.22	71.91 ± 8.35	0.64*
Sex	Female	48 (57.1%)	2 (25.0%)	50 (54.3%)	0.09**
	Male	36 (42.9%)	6 (75.0%)	42 (45.7%)	
Lens status	Phaco + PPV	46 (54.8%)	3 (37.5%)	49 (53.3%)	0.58***
	Phakic	5 (6.0%)	1 (12.5%)	6 (6.5%)	
	Pseudophakic	33 (39.3%)	4 (50.0%)	37 (40.2%)	
Preop IOP (mmHg)	mean ± SD	14.06 ± 2.88	11.50 ± 2.67	—	0.027†
Number of eyes		84	8	92	

*Student’s t-test. **Chi-square test. ***Fisher’s exact test. †Welch’s t-test. All ERMs were idiopathic. No patient had glaucoma or diabetes mellitus. Control group: eyes without postoperative CME. CME group: eyes that developed postoperative CME and received intravitreal dexamethasone implant. Preop IOP: preoperative intraocular pressure. SD: standard deviation

In the CME group, eight eyes from eight patients were included, with a mean age of 73.1 years. Most eyes were pseudophakic or had undergone combined phacoemulsification and PPV. The mean time interval between PPV and DEX implantation (Δt) and total follow-up time (FU) varied across patients, reflecting the natural history of CME onset and individual responses to prior topical treatment (Table 2).

**Table 2:** Demographic, clinical, and imaging characteristics of participants in the CME group

Sex	Age	Eye	Lens	Δt	FU	Pre Dex IOP	Post Dex IOP	Pre logMAR VA	Post logMAR VA	Pre Dex logMAR VA	Post Dex logMAR VA	Preop CMT	Postop CMT	Pre Dex CMT	Post Dex CMT
M	72	OS	PHACO + PPV	5	17	14	10	0.5	0	0.2	0	365.2	326.2	350.4	301.8
M	62	OS	Phakic	6	18	15	15	0.1	0	0.2	0	345	265.6	300.2	255
M	83	OS	Pseudophakic	5	18	15	11	0.4	0.8	0.8	0.6	387	338.5	364.6	336.4
F	76	OD	PHACO + PPV	6	18	12	17	0.7	0.4	1	0.6	422.4	325.4	386.9	292.7
M	71	OS	Pseudophakic	5	17	16	17	0.2	0	0	0	316.1	297.4	390	281
M	66	OS	PHACO + PPV	6	18	17	17	0.2	0.3	0.3	0.1	307	320.5	309.6	295
M	73	OS	Pseudophakic	3	15	19	16	0.8	0.6	0.6	0.2	542.2	300	428.2	289.7
F	82	OD	Pseudophakic	5	16	16	18	0.4	0.7	0.7	0.3	378	313.4	509	370

Abbreviations: logMAR: Logarithm of the minimum angle of resolution; PHACO: Phacoemulsification; PPV: Pars plana vitrectomy; Δt: Time in months from PPV to intravitreal dexamethasone implant; FU: Total follow-up time after PPV (months); IOP: intraocular pressure (mmHg); Pre Dex IOP: IOP before dexamethasone implant; Post Dex IOP: IOP after dexamethasone implant; Pre logMAR VA: Preoperative visual acuity; Post logMAR VA: Postoperative visual acuity; Pre Dex logMAR VA: Visual acuity before dexamethasone implant; Post Dex logMAR VA: Visual acuity after dexamethasone implant; Preop CMT: Preoperative central macular thickness (µm); Postop CMT: Postoperative central macular thickness (µm); Pre Dex CMT: Central macular thickness before dexamethasone implant (µm); Post Dex CMT: Central macular thickness after dexamethasone implant (µm); OD: Oculus dexter (right eye); OS: Oculus sinister (left eye)

### Intra-group comparisons of central macular thickness

In the control group, CMT significantly decreased after surgery, from 335.13 ± 37.28 μm preoperatively to 289.48 ± 22.87 μm postoperatively (mean difference: −45.65 μm; Cohen’s d = 1.39; 95% CI: −52.77 to − 38.53 μm; *p* < 0.001) (Table 3). Similarly, in the CME group, a significant reduction in CMT was observed following surgery, from 382.86 ± 74.59 μm to 310.88 ± 22.86 μm (mean difference: −71.99 μm; Cohen’s d = 0.94; *p* = 0.033). Furthermore, DEX implantation resulted in an additional significant reduction in CMT, from 379.86 ± 67.13 μm before implantation to 302.70 ± 35.34 μm after treatment (mean difference: −77.16 μm; Cohen’s d = 1.56; 95% CI: −109.18 to − 45.14 μm; *p* = 0.003). Non-parametric analyses (Wilcoxon signed-rank test) yielded consistent results for CME group comparisons.

**Table 3:** Intra-group comparisons of macular thickness

Group	Comparison	*n*	Pre (mean ± SD)	Post (mean ± SD)	Mean diff.	t	*p*-value
Control	Surgery	84	335.13 ± 37.28	289.48 ± 22.87	-45.65	12.714	< 0.001
CME group	Surgery	8	382.86 ± 74.59	310.88 ± 22.86	-71.99	2.645	0.033
CME group	Dexamethasone	8	379.86 ± 67.13	302.70 ± 35.34	-77.16	4.424	0.003

Values are expressed as mean ± SD (µm). A negative mean difference indicates reduction in CMT. Paired t-test for within-group comparisons; non-parametric (Wilcoxon signed-rank) results were consistent. “Surgery” = preoperative vs. postoperative; “Dexamethasone” = pre-implant vs. post-implant. CME: cystoid macular edema; SD: standard deviation

### Between-group comparisons of central macular thickness

At baseline, there was no statistically significant difference in CMT between groups (334.48 ± 36.81 μm vs. 382.86 ± 74.59 μm; *p* = 0.111). Following surgery, the CME group exhibited significantly higher CMT compared to controls (310.88 ± 22.86 μm vs. 289.48 ± 22.87 μm; *p* = 0.034) (Table 4). A marked and statistically significant difference was observed when comparing postoperative CMT in controls with pre-DEX values in the CME group (379.86 ± 67.13 μm vs. 289.48 ± 22.87 μm; *p* = 0.007). Following DEX implantation, no statistically significant difference in CMT was found between groups (302.70 ± 35.34 μm vs. 289.48 ± 22.87 μm; *p* = 0.332), indicating a reduction of the between-group difference to a non-significant level after treatment.

**Table 4:** Between-group comparisons of central macular thickness

Comparison	Controls (mean ± SD)	*n*	CME group (mean ± SD)	*n*	t	*p*-value
Pre-op: Control vs. CME group	334.48 ± 36.81	84	382.86 ± 74.59	8	-1.814	0.111
Post-op: Control vs. CME group	289.48 ± 22.87	84	310.88 ± 22.86	8	-2.524	0.034
Control post-op vs. CME pre-DEX	289.48 ± 22.87	84	379.86 ± 67.13	8	-3.786	0.007
Control post-op vs. CME post-DEX	289.48 ± 22.87	84	302.70 ± 35.34	8	-1.037	0.332

Values are expressed as mean ± SD (µm). Welch’s t-test. CME: cystoid macular edema; Pre-op: before surgery; Post-op: after surgery; Pre-DEX: before dexamethasone implant; Post-DEX: after dexamethasone implant

### Intra-group comparisons of best-corrected visual acuity

In the control group, BCVA improved significantly after surgery, with mean logMAR values decreasing from 0.32 ± 0.26 to 0.04 ± 0.10 (mean difference: −0.28; Cohen’s d = 1.12; 95% CI: −0.34 to − 0.23; *p* < 0.001) (Table 5). In contrast, the CME group did not show a statistically significant BCVA improvement following surgery (0.41 ± 0.25 to 0.35 ± 0.33; mean difference: −0.06; *p* = 0.582). After DEX implantation, the CME group demonstrated a significant BCVA improvement, with logMAR values decreasing from 0.47 ± 0.35 to 0.22 ± 0.25 (mean difference: −0.25; Cohen’s d = 0.89; 95% CI: −0.37 to − 0.13; *p* = 0.002). Non-parametric analyses (Wilcoxon signed-rank test) yielded consistent results for CME group comparisons.

Between-group comparisons revealed no significant difference in BCVA at baseline (*p* = 0.363). Postoperatively, BCVA was significantly worse in the CME group compared to controls (0.35 ± 0.33 vs. 0.04 ± 0.10; *p* = 0.032) (Table 6). A more pronounced difference was observed when comparing postoperative controls with CME group eyes prior to DEX (0.47 ± 0.35 vs. 0.04 ± 0.10; *p* = 0.010). Following DEX implantation, BCVA in the CME group improved, and the between-group difference was no longer statistically significant (0.22 ± 0.25 vs. 0.04 ± 0.10; *p* = 0.078), indicating partial functional recovery. However, it should be noted that the absolute difference of approximately 0.18 logMAR between groups — potentially corresponding to nearly two lines of visual acuity — remains clinically meaningful, and the non-significant p-value reflects limited statistical power rather than demonstrated clinical equivalence.

**Table 5:** Intra-group comparisons of best-corrected visual acuity

Group	Comparison	*n*	Pre (mean ± SD)	Post (mean ± SD)	Mean diff.	t	*p*-value
Control	Surgery	84	0.32 ± 0.26	0.04 ± 0.10	-0.28	10.255	< 0.001
CME group	Surgery	8	0.41 ± 0.25	0.35 ± 0.33	-0.06	0.576	0.582
CME group	Dexamethasone	8	0.47 ± 0.35	0.22 ± 0.25	-0.25	5.000	0.002

Values are expressed as mean ± SD (logMAR). Lower values indicate better visual acuity. Paired t-test; non-parametric (Wilcoxon signed-rank) results were consistent. “Surgery” = preoperative vs. postoperative; “Dexamethasone” = pre-implant vs. post-implant. CME: cystoid macular edema

**Table 6:** Between-group comparisons of best-corrected visual acuity

Comparison	Controls (mean ± SD)	*n*	CME group (mean ± SD)	*n*	t	*p*-value
Pre-op: Control vs. CME group	0.32 ± 0.26	84	0.41 ± 0.25	8	-0.962	0.363
Post-op: Control vs. CME group	0.04 ± 0.10	84	0.35 ± 0.33	8	-2.662	0.032
Control post-op vs. CME pre-DEX	0.04 ± 0.10	84	0.47 ± 0.35	8	-3.519	0.010
Control post-op vs. CME post-DEX	0.04 ± 0.10	84	0.22 ± 0.25	8	-2.054	0.078

Values are expressed as mean ± SD (logMAR). Lower values indicate better visual acuity. Welch’s t-test. Pre-op: before surgery; Post-op: after surgery; Pre-DEX: before dexamethasone implant; Post-DEX: after dexamethasone implant. CME: cystoid macular edema. Total eyes = 92; Controls = 84; CME group = 8; Incidence = 8.7%

### Intra-group comparisons of intraocular pressure

In the control group, no significant IOP change was observed after surgery (14.06 ± 2.88 to 14.29 ± 3.06 mmHg; *p* = 0.576) (Table 7). In contrast, the CME group showed a significant IOP increase following surgery, rising from 11.50 ± 2.67 mmHg preoperatively to 15.25 ± 3.73 mmHg postoperatively (mean difference: +3.75 mmHg; *p* = 0.019). The lower baseline IOP in the CME group — which was significantly different from that of the control group (*p* = 0.027) — may have contributed to the proportionally greater postoperative IOP increment observed in these eyes, potentially reflecting a propensity for inflammatory-mediated trabecular meshwork dysfunction. No significant IOP change was observed after DEX implantation in the CME group (15.50 ± 2.07 to 15.12 ± 3.00 mmHg; *p* = 0.747).

**Table 7:** Intra-group comparisons of intraocular pressure (IOP)

Group	Comparison	*n*	Pre (mean ± SD)	Post (mean ± SD)	Mean diff.	t	*p*-value
Control	Surgery	84	14.06 ± 2.88	14.29 ± 3.06	+ 0.24	-0.561	0.576
CME group	Surgery	8	11.50 ± 2.67	15.25 ± 3.73	+ 3.75	-3.035	0.019
CME group	Dexamethasone	8	15.50 ± 2.07	15.12 ± 3.00	-0.38	0.336	0.747

Values are expressed as mean ± SD (mmHg). Paired t-test. “Surgery” = preoperative vs. postoperative; “Dexamethasone” = pre-implant vs. post-implant. IOP: intraocular pressure; CME: cystoid macular edema

## Discussion

Postoperative macular edema following PPV for ERM represents a clinically meaningful complication that may substantially limit functional recovery despite anatomically successful surgery. In the present study, the incidence of postoperative CME requiring treatment with intravitreal dexamethasone implant was 8.7%, a rate that falls within the range previously reported in the literature, which varies considerably depending on diagnostic criteria, imaging modality, and follow-up duration [17, 20]. Lee et al. reported that cystoid macular edema was detectable on OCT in a notable proportion of eyes undergoing ERM surgery, and that its presence was associated with worse postoperative visual outcomes [14] — a finding directly corroborated by our results. The variability in reported incidence across studies likely reflects differences in the definition of CME, the sensitivity of imaging technology employed, and the timing of postoperative assessments, reinforcing the need for standardized diagnostic criteria in future investigations.

It should be emphasized that the CME cases described in this study do not represent degenerative microcystic macular edema (MME). Two distinct pathophysiological mechanisms have been proposed to explain intraretinal cystic changes in the context of ERM surgery: an inflammatory mechanism, characterized by blood–retinal barrier breakdown and increased vascular permeability driven by surgical manipulation and cytokine release; and a degenerative mechanism, involving retrograde transsynaptic degeneration and Müller cell dysfunction [14, 23, 24]. The latter has been described in conditions beyond ERM — including glaucoma, optic neuritis, and chiasmal compression — where microcysts within the inner nuclear layer arise in the absence of primary retinal vascular disease, driven instead by retrograde neuronal loss [24]. Dysli et al. specifically demonstrated that patients with ERM develop features consistent with retrograde maculopathy following ILM peeling, suggesting that a degenerative component may be present even in the surgical ERM context [25]. The distinction between inflammatory CME and degenerative MME carries meaningful clinical implications, as the former represents a potentially reversible target for anti-inflammatory intervention while the latter may be refractory to corticosteroid treatment.

Several features of our CME cases are more consistent with an inflammatory mechanism, although we acknowledge that definitive mechanistic attribution is not possible without fluorescein angiographic documentation of active leakage, which was not obtained in this cohort. All cystic changes were de novo postoperative findings, with no preoperative intraretinal cysts in any of the eight eyes; the temporal relationship between surgery and CME onset is consistent with postoperative inflammation; glaucoma and other conditions associated with retrograde transsynaptic degeneration were explicitly excluded; and the strict diagnostic criteria applied required documented CMT increase relative to the post-peeling baseline, thereby excluding degenerative microcysts without evidence of progressive thickening. The absence of fluorescein angiographic confirmation has been acknowledged as a limitation.

A key observation in the present study was the absence of significant baseline differences between the control and CME groups in terms of age, visual acuity, and macular thickness, indicating that the development of postoperative CME could not be predicted by standard preoperative clinical variables alone. This finding is consistent with the current understanding of the pathophysiology of postoperative macular edema, in which surgical manipulation — rather than pre-existing patient characteristics — is the primary trigger for intraocular inflammation, cytokine release, and subsequent breakdown of the blood–retinal barrier [17, 18]. The role of inflammatory mediators in ERM-related macular pathology has been well characterized, with studies demonstrating that glial cell activation and upregulation of growth factors and cytokines contribute to both membrane formation and postoperative retinal changes [26, 27]. This mechanistic framework supports the rationale for anti-inflammatory intervention in the postoperative period.

From an anatomical standpoint, both groups demonstrated a significant reduction in central macular thickness following surgery, as expected after ERM removal and relief of tangential traction. However, a critical divergence emerged in the postoperative period: despite this initial anatomical improvement, the CME group maintained significantly higher macular thickness compared to controls. This residual thickening likely reflects the contribution of ongoing inflammatory and vascular permeability mechanisms not resolved by vitrectomy alone. A similar pattern has been observed in prior studies evaluating postoperative OCT findings in ERM surgery, where residual intraretinal fluid was associated with incomplete functional recovery [14–16]. 

The anatomical response to DEX implantation in the CME group was significant, with CMT reducing from 379.86 ± 67.13 μm to 302.70 ± 35.34 μm (*p* = 0.003), reducing the between-group difference to a non-significant level. These findings are consistent with prior case series and cohort studies reporting anatomical benefits of DEX implant in this setting [22–30]. Taney et al. described meaningful macular thickness reductions in eyes with persistent edema after ERM surgery treated with sustained-release dexamethasone, even in cases refractory to other interventions [29]. Similarly, Furino et al. reported anatomical improvement in eyes with refractory postoperative edema following macular pucker surgery [28]. However, given the small sample size (*n* = 8) and the absence of a sham-treated comparator arm, spontaneous resolution of CME over time cannot be excluded as a contributor to the observed improvement, and these findings should be interpreted with appropriate caution as hypothesis-generating rather than confirmatory evidence of DEX efficacy.

Another important aspect of the study was the detrimental effect of CME on visual function. In the CME group, despite a significant reduction in macular thickness after vitrectomy, no corresponding improvement in visual acuity was observed. The presence of intraretinal cystic spaces within the inner nuclear and outer plexiform layers may directly impair photoreceptor function and disrupt normal neural transmission pathways even in the presence of partial anatomical recovery. In our previous work, we demonstrated that postoperative microcystic macular edema was among the structural findings most strongly associated with limited retinal sensitivity on microperimetry after ERM surgery [15], further supporting the functional relevance of persistent intraretinal fluid.

DEX implantation was associated with significant BCVA improvement (0.47 ± 0.35 to 0.22 ± 0.25 logMAR; *p* = 0.002; Cohen’s d = 0.89). Following treatment, BCVA in the CME group was no longer statistically different from that of the control group (*p* = 0.078). However, the absolute difference of approximately 0.18 logMAR — potentially representing nearly two lines of visual acuity — remains clinically meaningful. This non-significant p-value should not be equated with clinical equivalence in the context of an underpowered comparison, and a clinically important visual acuity gap may persist in a subset of CME-affected eyes despite treatment. Chang et al. reported similar functional gains following DEX implantation for long-standing macular edema after ERM peeling surgery [31]. Hattenbach et al. also documented functional benefits of sustained-release steroid implants in eyes with postoperative edema after ERM removal [30]. Together, the present findings suggest that intravitreal dexamethasone implant may be beneficial in selected cases of postoperative CME; however, larger prospective studies are required to confirm these results.

Yang et al. reported that different types of intraretinal cystoid spaces in idiopathic ERM are associated with distinct clinical profiles and prognoses, underscoring the heterogeneity of this finding and the risk of treating all cystic changes as equivalent therapeutic targets [23]. Mahmoudzadeh et al., in a large cohort of 322 eyes, further demonstrated that OCT biomarkers — including the nature and location of intraretinal fluid — were independently associated with visual outcomes after PPV for ERM [32]. In the present study, the favorable anatomical and functional response to DEX implantation is consistent with a predominantly inflammatory mechanism, given the relatively short interval between surgery and CME diagnosis and the prior exclusion of conditions predisposing to degenerative cystic changes. Nevertheless, a subset of patients may harbor a mixed or predominantly degenerative etiology, for whom the expected benefit of corticosteroid treatment would be more limited.

Regarding intraocular safety, the postoperative IOP elevation observed in the CME group preceded DEX implantation and is therefore attributable to the surgical procedure itself. After DEX implantation, no additional significant IOP change was observed (*p* = 0.747), suggesting that the implant did not exacerbate the postoperative IOP response. This is reassuring, as corticosteroid-induced ocular hypertension is a recognized complication of intravitreal DEX implants [21, 22]. The Bellocq et al. EPISODIC study, which evaluated DEX implants for post-surgical macular edema including Irvine-Gass syndrome, also reported that IOP elevations associated with DEX were generally transient and responsive to topical treatment [33]. 

Comparisons with studies evaluating other corticosteroid formulations are informative. Chen et al. reported that corticosteroid use was associated with improved anatomical outcomes but yielded inconsistent functional results across studies [17]. Ahn et al. found that intraoperative triamcinolone acetonide did not significantly improve visual outcomes compared to surgery alone [18]. Yonekawa et al. suggested potential advantages of the sustained-release DEX formulation over triamcinolone [20]. In the present study, intraoperative triamcinolone was used uniformly across all 92 eyes; since CME developed in only 8 of these, triamcinolone use alone was insufficient to prevent postoperative CME in all cases, and its presence as a uniform variable does not confound between-group comparisons.

Several limitations of the present study merit careful consideration. The retrospective design introduces the possibility of selection bias, as only eyes requiring treatment were included in the CME group, and subclinical or self-resolving cases of postoperative CME may have been missed. The small sample size of the CME group (*n* = 8) substantially limits statistical power, increases the risk of type II error, and reduces the generalizability of the findings. The absence of a sham-treated or topical-treatment-only comparator arm means that spontaneous CME resolution cannot be excluded as a contributor to the observed improvements, and causal attribution to DEX cannot be established. The absence of fluorescein angiographic confirmation precludes definitive mechanistic attribution of cystic changes. The variability in DEX implantation timing (range: 3–6 months post-PPV) reflects the retrospective nature of the study rather than a predefined protocol, and whether earlier intervention would have produced superior outcomes cannot be determined. No multivariate analysis was performed due to the small CME group size. The study was conducted at a single center with a single surgeon, which, while advantageous for procedural consistency, may limit generalizability. Finally, the lack of longer follow-up data precludes conclusions about the durability of anatomical and functional recovery.

Notwithstanding these limitations, the present study provides real-world preliminary evidence suggesting that intravitreal dexamethasone implant may be a beneficial and well-tolerated option for postoperative CME following ERM surgery in selected cases. Future prospective studies with larger sample sizes, angiographic confirmation of CME, standardized definitions and treatment protocols, and longer follow-up durations are warranted to confirm these findings and to identify predictors of postoperative CME development and treatment response.

## Data Availability

The datasets generated and/or analyzed during the current study are not publicly available due to patient privacy restrictions but are available from the corresponding author upon reasonable request.

## Declarations

### Authors’ information

Leonardo Provetti Cunha, MD, PhD – Associate Professor of Ophthalmology at the Juiz de Fora Medical School, Federal University of Juiz de Fora; Head of the Retina and Vitreous Service at Hospital de Olhos Juiz de Fora; Professor in the Postgraduate Program, Department of Ophthalmology, University of São Paulo, Brazil. Manuela Ferreira Guimarães – Medical student, Faculdade de Ciências Médicas e da Saúde de Juiz de Fora, Brazil. Leandro Cabral Zacharias, MD, PhD – Professor in the Postgraduate Program, Department of Ophthalmology, University of São Paulo, Brazil. Rony Carlos Preti, MD, PhD – Professor in the Postgraduate Program, Department of Ophthalmology, University of São Paulo, Brazil. Luciana Virgínia Ferreira Costa-Cunha, MD, PhD – Head of the Cataract Service at Hospital de Olhos Juiz de Fora, Brazil. Mário Luiz Ribeiro Monteiro, MD, PhD – Full Professor, Department of Ophthalmology, University of São Paulo, Brazil.

## Abbreviations

ANVISA: Agência Nacional de Vigilância Sanitária
BCVA: Best-corrected visual acuity
CAAE: Certificado de Apresentação de Apreciação Ética
CEP: Comitê de Ética em Pesquisa
CME: Cystoid macular edema
CMT: Central macular thickness
DEX: Dexamethasone intravitreal implant
DONFL: Dissociated optic nerve fiber layer
DRIL: Disorganization of the retinal inner layers
ERM: Epiretinal membrane
ILM: Internal limiting membrane
IOP: Intraocular pressure
logMAR: Logarithm of the minimum angle of resolution
MME: Microcystic macular edema
OCT: Optical coherence tomography
OD: Oculus dexter (right eye)
OS: Oculus sinister (left eye)
Phaco: Phacoemulsification
PPV: Pars plana vitrectomy
PVD: Posterior vitreous detachment
SD: Standard deviation
SPSS: Statistical Package for the Social Sciences
SS-OCT: Swept-source optical coherence tomography
UFJF: Universidade Federal de Juiz de Fora
